# Exercise-Context Sleep Quality Scale (ECSQS): Validity and Reliability Study

**DOI:** 10.3390/healthcare14193265

**Published:** 2026-10-02

**Authors:** Servet Ozoruc, Sultan Yavuz Eroglu, Erdem Eroglu, Mustafa Can Koc, Mahmut Ulukan, Laurentiu-Gabriel Talaghir, Gabriel Marian Manolache, Daniel Mădălin Coja, Teodora Mihaela Iconomescu, Bogdan Sorin Olaru

**Affiliations:** 1 Faculty of Sport Sciences, Osmaniye Korkut Ata University, Osmaniye 80000, Türkiye; servet_ozoruc@hotmail.com; 2 Faculty of Sports Sciences, Mus Alparslan University, Mus 49250, Türkiye; sultan-yvz@windowslive.com (S.Y.E.); medreeroglu@hotmail.com (E.E.); 3 Faculty of Sports Sciences, Mersin University, Mersin 33343, Türkiye; 4 Faculty of Sports Sciences, Istanbul Gelisim University, Istanbul 34310, Türkiye; mulukan@gelisim.edu.tr; 5 Faculty of Physical Education and Sport, Dunarea de Jos University of Galati, 800008 Galati, Romania; gabriel.manolache@ugal.ro (G.M.M.); daniel.coja@ugal.ro (D.M.C.); bogdan.olaru@ugal.ro (B.S.O.)

**Keywords:** exercise, sleep, sleep quality, validity, reliability

## Abstract

Background: The purpose of this study is to develop a valid and reliable measurement tool to assess sleep quality and sleep perceptions in the context of exercise among adults who engage in regular exercise. Methods: In line with this aim, the Exercise-Context Sleep Quality Scale (ECSQS) was developed, and exploratory and confirmatory factor analyses were conducted on two independent samples. It was determined that the scale has a structure consisting of a total of 12 items and three factors (Perception of Exercise-Related Sleep, Sleep Hygiene and Environmental Factors, general Sleep Adequacy). The results of the exploratory factor analysis showed that the total explained variance was 56.8%, which is above the thresholds recommended for construct validity in the social sciences. Results: The findings of the confirmatory factor analysis indicated that the three-factor model exhibited a good level of fit to the data according to the χ^2^/df ratio, RMR, RMSEA and fit indices such as GFI, AGFI, NFI, IFI, TLI and CFI. In reliability analyses, Cronbach’s alpha coefficient was 0.83 for the first factor, and 0.65 and 0.64 for the second and third factors, respectively; for the total scale, Cronbach’s alpha and McDonald’s omega values were found to be 0.77. Discriminant validity was supported by the Fornell–Larcker criterion, while convergent validity received only partial support because AVE values were below 0.50. Composite reliability (CR) values were acceptable. Item discrimination analyses showed that all items had item–total correlations above 0.30, and that Cohen’s d effect sizes in comparisons of the lower and upper 27% groups were mostly at “large” or “very large” levels; these results indicate that the scale strongly distinguishes between individuals with low and high levels of sleep quality in the context of exercise. Conclusions: The findings demonstrate that the ECSQS can validly and sufficiently reliably measure sleep quality in regular exercisers through a three-dimensional structure, and that, as one of the first Turkish sleep quality scales specific to the exercise context, it fills an important measurement gap in the sports sciences and sleep science literature.

## 1. Introduction

In this study, sleep quality is conceptualized as a subjectively assessed dependent variable examined in relation to both environmental stimuli and exercise behavior. According to the conceptual model, sleep quality can be enhanced, on the one hand, by reducing adverse environmental factors (e.g., high noise levels, excessive or inappropriate light exposure) and, on the other hand, by promoting appropriate exercise habits (regular aerobic and/or resistance training of individually suitable intensity and timing). It is assumed that the intensity and characteristics of environmental stimuli may impair ease of sleep onset and sleep continuity, whereas balanced exercise habits may improve sleep quality by increasing physical readiness for sleep and strengthening psychological well-being [1,2]. However, engaging in high-intensity exercise close to bedtime is considered potentially detrimental for some individuals, as it may prolong sleep onset latency and exert a stimulating effect; accordingly, the exercise–sleep relationship is assumed to be conditioned by the intensity, duration, and timing of exercise [2,3]. Sleep quality itself is a multidimensional construct that encompasses perceived restfulness, ease of sleep onset, sleep continuity, and feeling refreshed upon awakening, rather than a single, purely physiological indicator [4]. From this perspective, individuals who regularly engage in exercise may experience qualitatively different sleep patterns depending on how their training is scheduled, how they recover after exercise, and which environmental conditions surround their sleep [2,4]. Yet, most widely used sleep questionnaires treat physical activity as a generic lifestyle factor and do not systematically differentiate between exercise-related contextual elements (e.g., exercise timing relative to bedtime, type of exercise performed, subjective recovery status), which creates an important gap in both sport sciences and clinical sleep research [1,2,5]. In the literature, physical activity and exercise are widely accepted to play a protective and enhancing role in sleep architecture. Regular exercise is known to increase slow-wave sleep (deep sleep), shorten sleep onset latency, and optimize overall sleep quality [2,3]. (In particular, aerobic exercise has been shown to produce marked positive effects on sleep quality by regulating circadian rhythm [6], while resistance training has also been identified as an effective strategy for increasing sleep efficiency and reducing insomnia symptoms [7]. However, the subjective reflections of exercise type (aerobic vs. resistance), intensity, and timing on individuals’ sleep perceptions remain among the current topics of debate. Existing instruments such as the Pittsburgh Sleep Quality Index (PSQI) and the Epworth Sleepiness Scale (ESS) primarily focus on global sleep complaints, daytime sleepiness, and general sleep hygiene factors, but they do not explicitly operationalize exercise-specific variables (e.g., acute post-exercise arousal, delayed onset muscle soreness, or training load) within their item structure [8,9]. Consequently, these tools offer limited sensitivity when sleep quality is evaluated in populations for whom exercise is a central, routine behavior, such as recreationally active adults, competitive athletes, or patients in exercise-based rehabilitation programs [2,4]. The Exercise-Context-Specific Sleep Quality Scale (ECSQS) was therefore conceptualized to bridge this gap by integrating exercise-related dimensions into a sleep quality assessment framework that remains compatible with contemporary psychometric standards [1,2]. On the other hand, environmental and behavioral factors that undermine sleep hygiene also play a role. Exposure to blue-light-emitting screens (phones, tablets, computers) in the evening is known to suppress melatonin secretion and make it harder to fall asleep [10]. In addition, environmental pollution factors such as noise and light can cause sleep fragmentation and prevent transitions into deeper stages of sleep [11]. Moreover, it has been reported that consuming high-calorie or heavy meals late after exercise keeps metabolic activity and body temperature elevated, disrupts the circadian clock mechanism, and impairs sleep quality [12]. In addition to late eating patterns, other behavioral and environmental factors—including irregular sleep schedules, excessive screen exposure before bedtime, caffeine intake, and heightened stress levels—have also been shown to negatively affect both the quality and quantity of sleep, further underscoring the multifactorial nature of sleep disturbances [12]. Given this multifactorial background, there is a need for instruments that do not merely catalogue general sleep complaints, but that also capture how exercise-related behaviors and recovery processes interact with environmental and behavioural sleep-disrupting factors in everyday life [2,4,5]. The present study therefore aimed to develop and validate a Turkish scale that evaluates sleep quality specifically in individuals who exercise, grounding item content in the exercise–sleep literature and contemporary measurement theory so as to yield a context-sensitive tool for research and practice in sport and health sciences [1,2,13]. Given the complex interplay of these factors, there was a need for a comprehensive and psychometrically sound instrument to assess behaviours and conditions that may compromise sleep. Therefore, the scale development process was carried out based on the principles recommended in the psychometric theory and structural equation modelling literature [13,14]. This approach ensured that the scale captured relevant dimensions of sleep-related behaviours and environmental influences while maintaining robust reliability and validity. First, current theoretical and empirical studies on the exercise-sleep relationship were reviewed, and an item pool was created by considering the multidimensional nature of sleep quality and exercise-specific contextual factors [2,4,5]. During item development, attention was paid to ensuring that dimensions such as subjective sleep perception, post-exercise recovery, and environmental/physiological conditions that disrupt sleep were represented in the scale. Convergent and discriminant validity, composite reliability, and average variance extracted were evaluated, and the Fornell–Larcker criterion showed that inter-factor relationships were theoretically supportive of the scale dimensions [15,16]. Internal consistency reliability was examined using Cronbach’s alpha and McDonald’s omega coefficients, and item-level discrimination was evaluated through Cohen’s d effect sizes and item-total correlations [13,17]. The risk of common method bias associated with self-report measures was examined using Harman’s single-factor test as recommended by Podsakoff et al.; the fact that the variance explained by the single-factor model remained below the critical threshold indicated that CMB was not a major concern [18]. Nevertheless, future studies are recommended to combine ECSQS with objective sleep measures obtained through actigraphy, polysomnography, and wearable technologies, so that the exercise-sleep interaction can be evaluated multimethodally through both subjective and objective indicators [2,19]. Although the significance of Mardia’s statistics under the multivariate normality assumption theoretically represents a limitation for maximum likelihood (ML) estimation, the sample size of 310 and the fact that the fit indices were at acceptable levels indicate that the ML method worked robustly for this data structure [20]. In future validation studies, the use of Bollen–Stine bootstrap and Satorra–Bentler corrected approaches will strengthen the robustness of the model fit and facilitate replication of the results across different samples [21,22]. In conclusion, ECSQS has been developed as a valid and reliable Turkish scale specific to the exercise context, evaluating sleep quality in individuals who exercise through a three-dimensional structure, and has been introduced into the literature as a new measurement tool that offers context-sensitive assessment opportunities in sport sciences, health sciences, and sleep research [1,2]. Most existing instruments (e.g., PSQI, ESS) assess general sleep quality but do not explicitly capture exercise-specific contextual factors such as exercise timing, type, and perceived post-exercise recovery.

## 2. Methods

### 2.1. Research Questions

Against this backdrop, the primary research questions for our study were as follows:

* Is the Exercise-Context Sleep Quality Scale (ECSQS) a valid measurement tool?
* Is the Exercise-Context Sleep Quality Scale (ECSQS) a reliable measurement tool?

### 2.2. Research Model

In this study, a quantitative research model employing a survey design was used. For the research group in the first study, the aim was to reveal the structure of the scale that resulted from the item pool created by defining the sleep quality domain of individuals who engage in regular exercise. The research group was selected using a convenience sampling method, taking ease of access into account [23]. The study included adults who reported regular exercise and were selected from Osmaniye province of Türkiye using a convenience sampling method. A total of 620 participants were divided into two independent samples of equal size (*n* = 310 for EFA and *n* = 310 for CFA). Regular exercise status was assessed using a Personal Information Form. The age range of the participants was determined as 18–55. EFA data were collected between 3 January 2026 and 13 March 2026. How many days a week do you exercise regularly? EFA participants responded with 1–2 days 30.3% (94), 3–4 days 41.6% (129), and 5 days or more 28.1% (87). CFA data were collected between 09.04.2026 and 09.05.2026. How many days a week do you exercise regularly? CFA responded as 33.3% (103) for 1–2 days, 40.6% (126) for 3–4 days, and 27.1% (84) for 5 days and over. Descriptive statistical information regarding the participants is presented below.

In Table 1, in the EFA sample 55.5% of the participants are male (*n* = 172) and 44.5% are female (*n* = 138), while in the CFA sample the gender distribution is more balanced (50.3% female, *n* = 156; 49.7% male, *n* = 154). In both samples, the vast majority of participants are single (EFA: 81.0%; CFA: 80.6%). In terms of educational status, undergraduate graduates constitute the majority in the EFA sample (80.6%), whereas in the CFA sample postgraduate graduates predominate (74.5%). When the most frequently performed type of exercise is examined, in the EFA sample the highest rate belongs to combined exercise (37.1%), followed by strength (32.6%) and aerobic (30.3%) exercises. In the CFA sample, the highest rate belongs to strength exercise (39.7%), followed by combined (35.2%) and aerobic (25.2%) exercises.

### 2.3. Procedure for Scale Development

#### Interviews and Literature Review

In the initial phase of the scale development process, face-to-face interviews were conducted with five faculty members from different departments of the Faculty of Sport Sciences (two professors in sport psychology, one associate professor in measurement and evaluation, one assistant professor in exercise physiology, and one lecturer in coaching education). These experts were selected based on their academic qualifications and prior experience in scale development and psychometrics. Each expert was asked to evaluate the relevance, clarity, and representativeness of the proposed items using a 4-point rating scale (1 = not relevant, 2 = somewhat relevant, 3 = quite relevant, 4 = highly relevant).

The evaluation procedure involved independent ratings, after which the researcher calculated the Content Validity Index (CVI) for each item and the overall scale. Items with CVI values below 0.80 or Content Validity Ratio (CVR) values below the Lawshe critical threshold were either revised or deleted. As a result of this process, four items were removed due to low relevance scores, and several items were reworded for clarity. The final item pool consisted of 17 items, which were then subjected to exploratory factor analysis.

### 2.4. Obtaining Expert Opinion (Content Validity)

The initial item pool consisted of 21 statements developed based on the theoretical framework and expert feedback. During the first stage, items with low corrected item–total correlations (<0.30) and overlapping content were removed, resulting in a 17-item version. In the second stage, exploratory factor analysis (EFA) was conducted, and items with factor loadings below 0.40 or cross-loadings on multiple factors were excluded. This process yielded the final 12-item scale. At each stage, decisions were guided by both statistical criteria (item–total correlations, factor loadings, communalities) and theoretical considerations (conceptual clarity, relevance to the construct). Thus, the reduction from 21 to 17 and finally to 12 items was systematically based on psychometric evidence and conceptual alignment.

### 2.5. Data Collection Tool

*Personal Information Form:*

A demographic information form developed by the researcher was used to determine the participants’ gender, marital status, educational status, and type of exercise.

*Exercise-Context Sleep Quality Scale ECSQS):*

For use in the study, the initial version of the “Exercise-Context Sleep Quality Scale” consisted of 17 items. The scale, applied to measure Sleep Quality in the Context of Exercise among individuals who exercise regularly, was rated on a 5-point Likert scale.

*Administration of the Scale:*

Data were collected via an online survey (Google Forms). In the form, participants were informed about the scientific nature of the study, and it was stated that providing objective responses would contribute to obtaining accurate and reliable results. In addition, voluntary informed consent was obtained from participants prior to the study.

*Compliance with Ethical Rules:*

To evaluate the ethical appropriateness of the study, the Health Sciences Research Ethics Committee of Osmaniye Korkut Ata University, at its meeting numbered 5 and dated 23 May 2025, unanimously decided to grant an Ethics Committee Approval Certificate to the study registered under number E.230784.

### 2.6. Analyzing the Data

The statistical analyses of the research data were carried out using the IBM SPSS Statistics 22.0 and AMOS 24.0 software packages. Before starting the analyses, the data set was examined in terms of missing data, outliers, and normality assumptions. Missing data were excluded from the analyses using the listwise method. To assess whether the variables showed a normal distribution, skewness and kurtosis values were examined; values within the ±2 range were considered to indicate that the normality assumption was met. Accordingly, parametric statistical methods were used in the analyses. To determine the construct validity of the scale, Exploratory Factor Analysis (EFA) was first conducted. Before EFA, the suitability of the sample for factor analysis was evaluated using the Kaiser–Meyer–Olkin (KMO) coefficient and Bartlett’s Test of Sphericity. In the initial analyses, principal component analysis (PCA) with Varimax rotation was employed. However, given that the reported factor correlations were moderate, a common-factor extraction method was deemed more appropriate. Therefore, the analyses were rerun using principal axis factoring (PAF) with an oblique rotation (Promax), which allows for correlated factors. This approach provided a more theoretically consistent solution and yielded factor loadings and correlations that better reflect the latent structure of the ECSQS. The decision to adopt PAF with oblique rotation was based on both statistical evidence and the conceptual expectation that dimensions of sleep quality in the context of exercise are interrelated.

Factors with eigenvalues greater than 1 were taken into account, and a minimum factor loading of 0.30 for items was adopted as a criterion. In addition, parallel analysis was conducted to determine the number of factors to retain. The results indicated that the first three observed eigenvalues (3.73, 1.70, 1.38) exceeded the corresponding random eigenvalues generated by the procedure, thereby confirming the retention of three factors. To confirm the factor structure obtained from EFA, Confirmatory Factor Analysis (CFA) was performed. In CFA, model fit was evaluated using the fit indices χ^2^/df, GFI, NFI, RFI, IFI, TLI, CFI, and RMSEA. Model fit values within acceptable limits were considered as evidence supporting the construct validity of the scale. The reliability of the scale was assessed using Cronbach’s alpha internal consistency coefficient. To determine the discriminatory power of the scale, the scores of the lower 27% and upper 27% groups formed according to the total scale scores were compared using an independent samples *t*-test. In all statistical analyses, the level of significance was set at *p* < 0.05. The findings obtained from these analyses were evaluated together to demonstrate that the scale is a valid and reliable measurement tool. Convergent and discriminant validity, composite reliability, and average 83 variance extracted were evaluated, and the Fornell–Larcker criterion showed that inter-84 factor relationships were theoretically supportive of the scale dimensions [15,16]. Internal 85 consistency reliability was examined using Cronbach’s alpha and McDonald’s omega 86 coefficients, and item-level discrimination was evaluated through Cohen’s d effect sizes 87 and item-total correlations Cohen’s d effect sizes were interpreted according to Cohen (1988) classification [13,15,17]. The risk of common method bias associated with self-88 report measures was examined using Harman’s single-factor test as recommended by 89 Podsakoff et al.; the fact that the variance explained by the single-factor model remained 90 below the critical threshold indicated that CMB was not a major concern [18].

### 2.7. Common Method Bias Analysis

To assess common method bias, Harman’s single-factor test was conducted. The results showed that the first factor accounted for 18.6% of the variance, which is well below the critical threshold of 50%. This indicates that common method bias is not a major concern in the present study. However, it should be noted that Harman’s test alone does not definitively rule out common method bias. Therefore, future research is encouraged to employ multi-method approaches (e.g., objective measures, longitudinal designs) to further minimize potential bias.

## 3. Results

In the exploratory factor analysis, a three-factor solution was obtained, explaining 56.8% of the total variance (see Table 2). Factor loadings ranged between 0.65 and 0.81, supporting the construct validity of the scale.

Confirmatory factor analysis results indicated that the three-factor model provided an acceptable fit to the data. The goodness-of-fit indices are presented in Table 4, showing that χ^2^/df, RMSEA, and CFI values were within recommended thresholds.

All items loaded significantly on their respective factors, with values above 0.50, which provides partial support for convergent validity. However, AVE values remained below 0.50, which should be considered a limitation. Composite reliability (CR) and average variance extracted (AVE) values are shown in Table 6. While CR values reached acceptable levels, AVE values remained below 0.50, which should be considered a limitation in terms of convergent validity.

Finally, the measurement model tested in the confirmatory factor analysis is illustrated in Figure 2. The diagram demonstrates the three-factor structure and the significant associations between items and their latent constructs.

### 3.1. Exploratory Factor Analysis (EFA)

Before the exploratory factor analysis, the suitability of the data set for factor analysis was evaluated using the Kaiser–Meyer–Olkin (KMO) measure of sampling adequacy and Bartlett’s test of sphericity. The KMO value of 0.82 indicated that the sample size was at an excellent level for factor analysis. Bartlett’s test of sphericity was significant (χ^2^ (105) = 1146.32, *p* < 0.001), showing that there was sufficient correlation among the items to conduct factor analysis.

As a result of the analysis, three factors with eigenvalues greater than 1 were obtained. The first factor explained 31.07% of the total variance, the second factor explained 14.23%, and the third factor explained 11.50%; together, the three factors accounted for 56.8% of the total variance. Since in the social sciences a total explained variance above 50% is considered adequate, these results support the construct validity of the scale.

The first factor was named “perception of exercise-related sleep”, and the factor loadings of the items in this factor ranged from 0.65 to 0.81. The second factor was labeled “sleep hygiene and environmental factors”, with factor loadings ranging from 0.71 to 0.75. The third factor was named “General Sleep Adequacy”, and the factor loadings of the items in this dimension ranged from 0.65 to 0.80.

**Figure 1:**
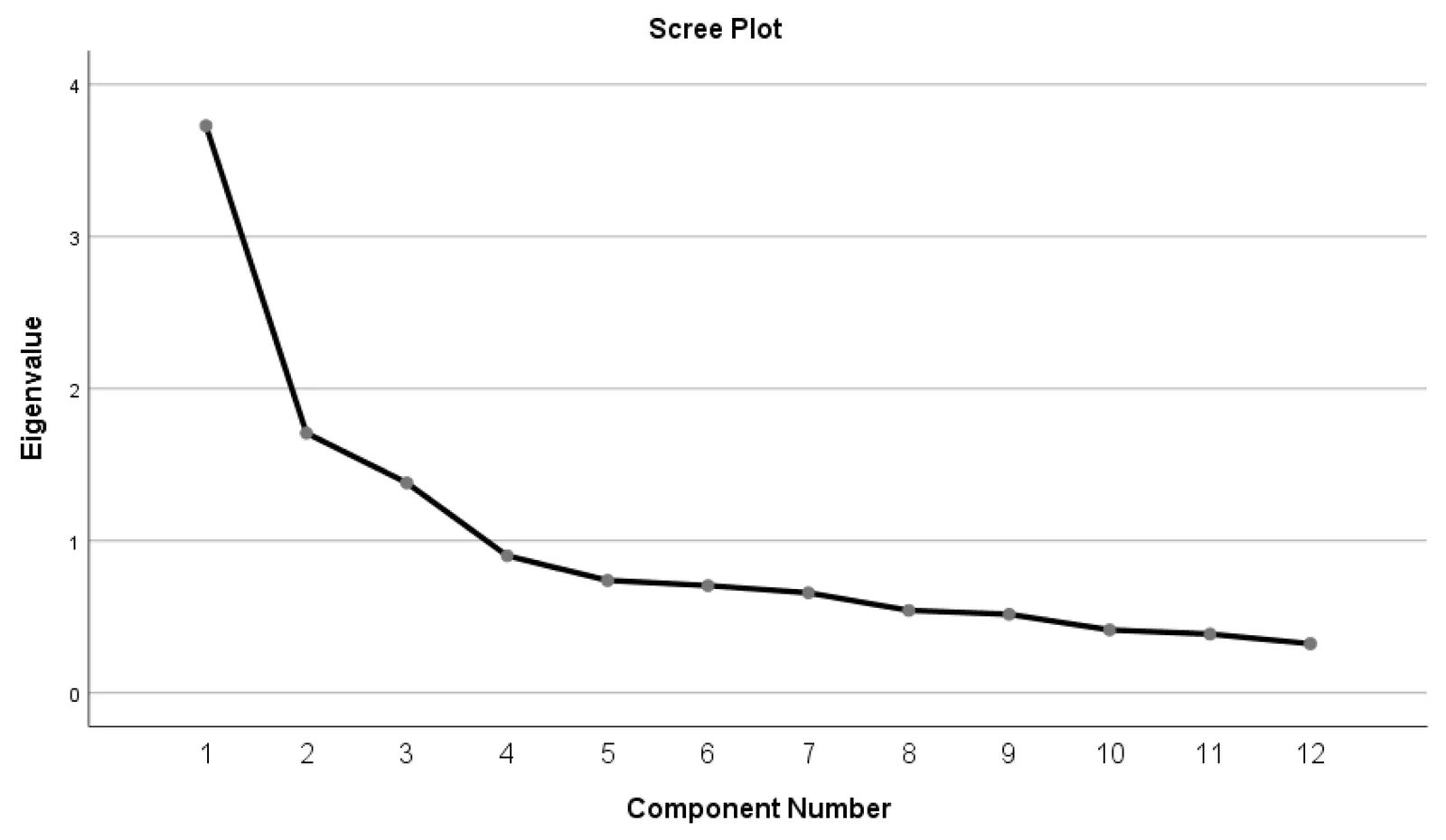
Factor Accumulation Graph.

The common variance (h^2^) values of the items ranged from 0.44 to 0.69, indicating that all items were adequately explained by the common factor structure. The correlation coefficients between the factors ranged from 0.472 to 0.547, showing that the subscales were moderately correlated with each other but conceptually distinguishable. This finding supports the use of an oblique rotation method (Promax), which assumes a certain level of correlation among the factors. In addition to eigenvalues greater than 1 and the scree plot, a parallel analysis was conducted using Jamovi (2.3.28.0). The results indicated that the first three observed eigenvalues exceeded those obtained from randomly generated data, thereby confirming the retention of three factors (Figure 1). This provides stronger empirical support for the three-factor solution.

When Table 3 is examined, the reliability analyses support the three-factor structure of the scale. Factor 1 (F1) shows high reliability (α = 0.83, ω = 0.83). The corrected item–total correlations of the items range between 0.52 and 0.71, indicating that all items make a strong contribution to the scale. Therefore, none of the items in Factor 1 were removed from the scale. For Factor 2 (F2), Cronbach’s alpha and McDonald’s omega were found to be 0.65. Therefore, we recommend using the total scale score as the primary indicator, and treating subscale scores as exploratory until further refinement and replication studies are conducted. The item–total correlations (0.37–0.52) show that the items contribute to the scale. Accordingly, it was deemed appropriate to retain the items in Factor 2. In addition, examination of Cronbach’s alpha values showed that removing item 6 would slightly increase the reliability of the scale (0.64); however, this increase is limited. Therefore, instead of removing item 6 from the scale, it was considered appropriate to keep it in the model.

For Factor 3 (F3), Cronbach’s alpha and McDonald’s omega were found to be 0.64. Although these values are borderline, they are within the acceptable range. The item–total correlations (0.42–0.48) indicate that the items contribute to the scale. For this reason, it was concluded that the items in Factor 3 should also not be removed from the scale. For the total scale (12 items), Cronbach’s alpha and McDonald’s omega were found to be 0.77. These values indicate that the scale is generally reliable. Since all items were collected from the same participants using a self-report method during the data collection process, the possibility of common method bias (CMB) was evaluated using Harman’s single factor test [18].

### 3.2. Confirmatory Factor Analysis (CFA)

**Figure 2:**
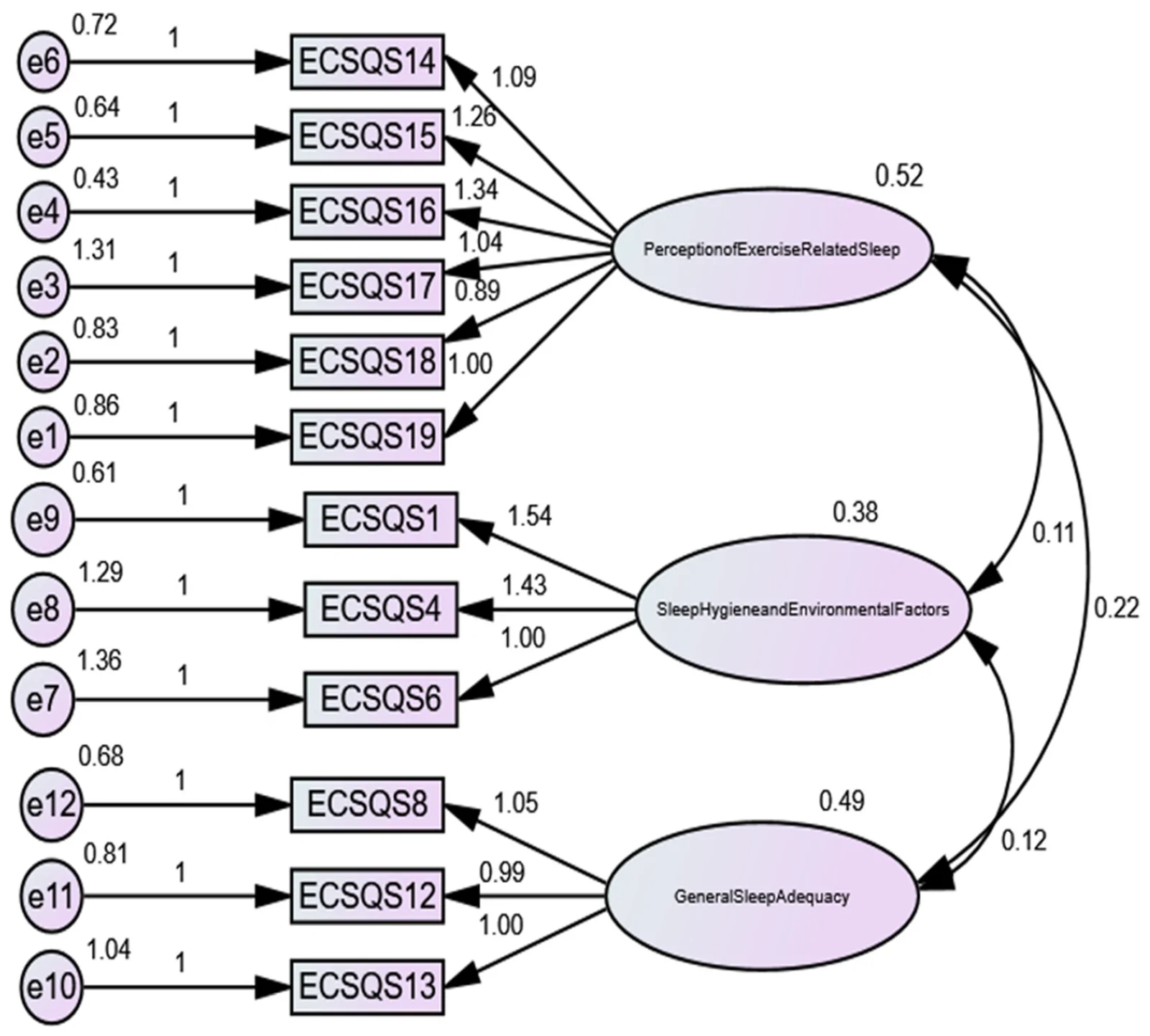
CFA Diagram.

In order to confirm the three-factor structure obtained as a result of the Exploratory Factor Analysis, Confirmatory Factor Analysis (CFA) was conducted. Within the scope of the analysis, a measurement model consisting of three latent variables, namely F1, F2, and F3, was tested. The standardized path coefficients and factor structure for the specified model are presented in Figure 2.

Because confirmatory factor analysis was conducted using the Maximum Likelihood (ML) method, the assumption of multivariate normality was evaluated using Mardia’s [19] multivariate skewness and kurtosis statistics. The analysis showed that both the multivariate skewness and multivariate kurtosis statistics were significant, indicating that the data set did not meet the assumption of exact multivariate normality. As noted in the literature, when the sample size is sufficiently large, maximum likelihood estimation is relatively robust to moderate deviations from multivariate normality [20]. In this study, considering that the CFA sample consisted of 310 participants and that the model fit indices were at an acceptable level, it was assumed that the deviation from multivariate normality did not substantially affect the model results. Nevertheless, for future validation studies, it is recommended to use estimation methods that are more robust to deviations from the normality assumption, such as Bollen–Stine bootstrap or Satorra–Bentler adjusted estimation [21,22].

In Table 4, the fit of the measurement model was evaluated using absolute, incremental, and parsimony-based goodness-of-fit indices. The results indicated that the model showed a good fit to the data. The chi-square/degrees of freedom ratio (χ^2^/df = 2.15) being below 5 indicates a high level of model fit. Similarly, the RMR value (0.057) was within acceptable limits, and the RMSEA value (0.06) indicated acceptable fit. When the incremental fit indices were examined, the GFI (0.94), AGFI (0.91), NFI (0.94), IFI (0.94), TLI (0.92), and CFI (0.94) values were found to be above the recommended threshold values.

When the standardized factor loadings were examined, it was determined that all items represented their respective latent variables at a significant level (*p* < 0.001). The standardized loadings of the items on the first factor ranged from 0.55 to 0.83, on the second factor from 0.57 to 0.77, and on the third factor from 0.56 to 0.67. The fact that all standardized factor loadings are above the recommended 0.50 threshold indicates that the items adequately represent their respective factors. Table 5 shows the fit indices of the proposed three-factor model compared to the independent and saturated models. As expected, the saturated model produced perfect fit values, while the independent model showed poor fit. This finding indicates that the three-factor model represents the data significantly better and supports the construct validity of the scale.

As shown in Table 6, the standardized loadings of the three-factor model are significant (*p* < 0.001) and range from 0.55 to 0.83. For Factor 1—Perception of Exercise-Related Sleep—the CR value was 0.82 and the AVE value was 0.40. These results indicates high internal consistency, but convergent validity is only partially supported. For Factor 2—Sleep Hygiene and Environmental Factors—the CR value was 0.80 and the AVE value was 0.41; these values are at the borderline of acceptability and reliability is acceptable, but convergent validity remains limited and should be reported as a limitation. For Factor 3—General Sleep Adequacy—the CR value was 0.74 and the AVE value was 0.39; while these results indicate acceptable internal consistency, but convergent validity is weak due to AVE values below 0.50. According to the Fornell–Larcker criterion, the √AVE value for each factor was found to be higher than the inter-factor correlations (F1–F2 = 0.25; F1–F3 = 0.43; F2–F3 = 0.28). This finding supports the discriminant validity of the three factors. Furthermore, the fact that the item–total correlations are above 0.30 and that Cohen’s d values range from 1.20 to 1.80, indicating very strong item discrimination, demonstrates that the scale’s items effectively differentiate between individuals with high and low scores. Composite Reliability (CR) and Average Variance Extracted (AVE) values were calculated using the standardized factor loadings reported in Table 6. These indices are not presented directly in the table but were derived from the loadings to provide additional evidence of reliability and validity. Composite reliability (CR) values ranged between 0.74 and 0.82. While two factors exceeded the recommended threshold of 0.70, one factor was slightly below but still within an acceptable range, suggesting adequate internal consistency) [14]. Average variance extracted (AVE) values ranged between 0.39 and 0.41, which are below the conventional 0.50 cutoff [16]. Although these indicate limited convergent validity, the factor loadings were all significant (*p* < 0.001) and above 0.50, which provides partial evidence for convergent validity. Overall, the reliability of the scale can be considered acceptable, but convergent validity should be interpreted cautiously due to AVE values below 0.50 (Table 7).

### 3.3. Scoring the Scale

In the Exercise-Context Sleep Quality Scale, the scores given to the items are summed up. The lowest score on the scale is 12, and the highest is 60. A higher score indicates a higher level of sleep quality in the context of exercise for the participants.

The Exercise-Context Sleep Quality Scale (ECSQS) consists of 12 items rated on a 5-point Likert scale. Factor 1 (“Perception of Exercise-Related Sleep”) includes six items, Factor 2 (“Sleep Hygiene and Environmental Factors”) includes three items, and Factor 3 (“General Sleep Adequacy”) includes three items. The total score ranges from 12 to 60, with higher scores indicating better overall sleep quality in the context of exercise. As shown in Table 8, descriptive statistics demonstrate that participants reported moderate levels of sleep quality across all factors, with Factor 1 showing the greatest variability and Factor 3 the most consistent responses.

The total score of the scale was calculated so that higher values indicate better sleep quality. Negatively worded items (ECSQS8, ECSQS12, and ECSQS17) were reverse scored. After these items were reversed, all items were summed to get the total score of the scale.

Items with corrected item–total correlations below 0.30, factor loadings below 0.40, or cross-loadings on multiple factors were deleted. Decisions were based on both statistical criteria and theoretical considerations (Table 9).

## 4. Discussion

In this study, the psychometric properties of the Exercise-Context Sleep Quality Scale (ECSQS), developed to evaluate sleep quality in the context of exercise among individuals who engage in regular exercise, were examined. Exploratory and confirmatory factor analyses conducted on two independent samples showed that the scale has a three-factor, 12-item structure, and that this structure is statistically well supported. The total explained variance of 56.8% exceeds the commonly used acceptable thresholds (50% and above) in scale development studies in the social sciences, thereby supporting construct validity [15]. The first factor, “Perception of Exercise-Related Sleep,” captures the reflections of subjective benefits related to exercise on sleep quality. Factor loadings in this dimension range between 0.55 and 0.83, and the Cronbach’s alpha coefficient of 0.83 indicates high internal consistency of the subscale. In the second factor, “Sleep Hygiene and Environmental Factors” (0.57–0.77 factor loadings; α = 0.65), and in the third factor, “General Sleep Adequacy” (0.56–0.67 factor loadings; α = 0.64), the reliability coefficients are at the borderline but are considered acceptable for newly developed scales in the social sciences literature [8,14]. Cronbach’s alpha and McDonald’s omega values of 0.77 for the total scale show that the scale has satisfactory internal consistency at the overall level. Although the total scale demonstrated acceptable reliability, the internal consistency of Factors 2 and 3 was relatively low (α = 0.65; ω = 0.64). These values indicate that the subscales should be interpreted with caution, as they may not capture the constructs as robustly as desired. This limitation suggests that the use of the total score is more reliable than relying on individual subscale scores, and future studies should aim to refine items or expand the item pool to improve the reliability of these dimensions. The development of ECSQS responds to the lack of a self-report instrument specific to the exercise context, given that studies evaluating the relationship between exercise and sleep in the literature largely rely on general sleep scales (e.g., Pittsburgh Sleep Quality Index, PSQI; Epworth Sleepiness Scale) [4,5]. Many studies examining the effects of exercise on sleep have reported improvements in sleep duration and some sleep parameters among exercising individuals, but no consistent advantage in subjective sleep quality scores [2,19]. For example, in a study on adolescent athletes, no clear dose–response relationship was found between exercise intensity and subjective sleep quality; however, it was reported that the sleep duration of exercising groups was approximately 50 min longer than that of the control group [9]. These findings indicate that the interaction between exercise and sleep should be evaluated not only in terms of duration and objective parameters, but also through subjective perception and contextual factors. The fact that the first factor of ECSQS brings together the effect of exercise on sleep, the second factor subjective sleep perception, and the third factor conditions that impair/improve sleep under the same umbrella meets this multidimensional need, particularly for exercise-engaged samples. Furthermore, the development of the ECSQS aligns with findings from recent meta-analyses and systematic reviews highlighting the inadequacy of assessing the exercise–sleep relationship solely through global sleep quality indices. Kredlow et al. reported that both acute and chronic effects of exercise have a moderate impact on sleep—specifically on parameters such as sleep latency, total sleep time, and efficiency—yet do not yield consistent or substantial effects on subjective sleep quality scores [2]. Similarly, Buman and King emphasized that while improvements in sleep duration and satisfaction are observed in individuals who exercise, this relationship is better explained by contextual factors (such as exercise timing, type, intensity, and sleep hygiene behaviors) [19]. Reports from the American College of Sports Medicine (ACSM) and relevant expert groups also recommend the combined use of subjective and objective indicators, as well as the consideration of contextual determinants (e.g., environmental noise, light, physical fatigue, exercise timing), when evaluating the relationship between sleep and exercise. In this context, the ECSQS—with its exercise-specific focus on the sleep context and its three-dimensional structure (exercise-related sleep perception, sleep hygiene/environmental factors, and general sleep adequacy)—can be said to reflect the contextual and multi-level assessment approach increasingly advocated in the literature [1,2]. Findings from the confirmatory factor analysis show that the three-factor model demonstrates a good level of fit with the data. A χ^2^/df ratio of 2.15, RMR and RMSEA values within acceptable limits, and incremental fit indices such as GFI, AGFI, NFI, IFI, TLI, and CFI above 0.90 are consistent with fit criteria recommended in the structural equation modeling literature [20,24]. Mardia’s coefficients showed that there were violations of multivariate normality. Even though the maximum likelihood estimation gave acceptable fit indices, this issue was tackled by repeating the confirmatory factor analysis in AMOS with bootstrap procedures using 200 resamples. The bootstrap method produced robust standard errors and bias-corrected confidence intervals, which helped make the parameter estimates more reliable. Importantly, the bootstrap results confirmed that the three-factor model was stable, with fit indices (χ^2^/df = 2.15, CFI = 0.94, TLI = 0.92, RMSEA = 0.06, SRMR = 0.069) staying within acceptable limits. Confirmatory factor analysis results indicated that the three-factor model provided an acceptable fit to the data, with standardized factor loadings ranging from 0.55 to 0.83, all exceeding the conventional 0.50 threshold, which provides partial evidence for convergent validity. However, AVE values remained below 0.50, indicating that convergent validity is limited. Reliability analyses showed that corrected item-total correlations ranged from 0.37 to 0.71, with Cronbach’s alpha values of 0.83 for Factor 1, 0.65 for Factor 2, and 0.64 for Factor 3, and 0.77 for the total scale, alongside parallel McDonald’s omega values. While the total scale demonstrated satisfactory internal consistency, the relatively lower reliability of Factors 2 and 3 suggests that subscale scores should be interpreted with caution, and future studies are advised to refine or expand the item pool to strengthen reliability and validity. Furthermore, Composite reliability (CR values between 0.74 and 0.82) and AVE values (0.40, 0.41, 0.39) being borderline but acceptable, and each factor’s √AVE value being greater than inter-factor correlations according to the Fornell–Larcker criterion, show that both convergent and discriminant validity have been indicated partial support for convergent validity [15,16]. The average variance extracted (AVE) values for the three factors (0.40, 0.41, and 0.39) fell below the recommended threshold of 0.50, indicating that the constructs may not demonstrate strong convergent validity. While these values are sometimes considered marginally acceptable in early-stage scale development, they highlight a limitation of the present study. The low AVE values may be attributed to the broad scope of the items, which encompass dimensions such as exercise type, environmental factors, and general sleep, resulting in the factors not fully accounting for the variance. Nevertheless, the scale provides a holistic assessment of sleep quality within the context of exercise. Therefore, the convergent validity evidence should be interpreted with caution, and future research should aim to refine the item pool or expand the number of items to strengthen the validity of these dimensions. Item discrimination findings indicate that the scale strongly differentiates between individuals with low and high scores on the construct being measured. In comparisons of the upper–lower 27% groups, all items had item–total correlations above 0.30, and Cohen’s d effect sizes were mostly in the “large” (≥0.80) and “very large” (≥1.20) categories, demonstrating that the ECSQS items exhibit consistent relationships with the total score and provide strong practical discrimination [13,17]. In particular, d values between 1.20 and 1.80 for exercise-related items show that individuals with low and high levels of exercise-context sleep quality differ markedly in terms of their scale scores. When the demographic characteristics of the sample are examined, it is seen that the study consists of adult individuals who regularly exercise in the province of Osmaniye, that single participants are in the majority in both samples, and that undergraduate–postgraduate education levels are predominant. This means that the initial validation of the scale was performed on a relatively well-educated group already engaged in exercise; therefore, additional studies are needed to generalize the results to different regions, socioeconomic levels, and clinical populations. Nonetheless, considering that studies investigating sleep and quality of life in individuals who regularly exercise have similarly been conducted with samples displaying high levels of physical activity [1,2], ECSQS can be considered a suitable starting point for use in research in this field. Since the measurement method of this study is based on a self-report form, the risk of common method bias (CMB) was evaluated using Harman’s single-factor test, and the proportion of total variance explained by the single-factor model being below the critical threshold indicates that CMB is not a major issue [18]. However, measuring all variables at the same time and with the same instrument should be supported in future research through multi-method designs (e.g., sleep diaries, actigraphy, objective sleep measures); this approach is in line with the increasing trend toward multilevel analyses of the exercise–sleep relationship in recent years [2,19]. Although significant Mardia statistics in the multivariate normality assumption theoretically pose a limitation for maximum likelihood (ML) estimation, the sample size of 310 and the fit indices being at acceptable levels indicate that the ML method is robust for this data structure [20]. Nevertheless, using Bollen–Stine bootstrap or Satorra–Bentler corrected methods in future validation studies will enhance the robustness of the results [21,22]. As a result of this study, the Exercise-Context Sleep Quality Scale (ECSQS) that was developed has been introduced as a valid and reliable measurement tool that evaluates sleep quality in individuals who exercise regularly through a three-dimensional structure. The overall reliability coefficients of the scale are at an acceptable level (α = 0.77, ω = 0.77), but the reliability values obtained for Factor 2 (Sleep Hygiene and Environmental Factors) and Factor 3 (General Sleep Adequacy) (0.64–0.65) are at a borderline acceptable level. Therefore, it is recommended that total scores be primarily considered in the general use of the scale, while subdimension scores should be evaluated in an exploratory manner. The confirmatory factor analysis results obtained show that the three-factor model is compatible with the data. However, the limited reliability values for Factors 2 and 3 suggest that in future studies, these dimensions of the scale should be tested on a larger sample and, if necessary, the item structure should be reviewed. Overall, the ECSQS fills an important gap in the Turkish literature for measuring sleep quality in the context of exercise, but it should be interpreted carefully, keeping in mind the limitations of the sub-dimensions. Overall, ECSQS is one of the first Turkish sleep quality scales specific to the exercise context that combines the effect of exercise on sleep, the individual’s subjective sleep perception, and environmental/physiological general sleep adequacy within a single measurement tool. In this respect, it has the potential to contribute to both the sports sciences and sleep science literature by enabling more nuanced testing of the relationships between sleep quality and exercise type, frequency, intensity, and duration; moreover, future studies are advised to combine ECSQS with objective sleep measures obtained via actigraphy, polysomnography, and wearable technologies so that the exercise–sleep interaction can be evaluated multimethodally through both subjective and objective indicators [2,19], and, although the significance of Mardia’s statistics under the multivariate normality assumption theoretically represents a limitation for ML, the sample size and satisfactory fit indices show that ML worked robustly in this dataset [20] with the planned use of Bollen–Stine bootstrap and Satorra–Bentler corrected approaches expected to enhance model fit robustness and replicability [21,22], leading to the conclusion that ECSQS has been developed as a valid and reliable Turkish scale specific to the exercise context, evaluating sleep quality among exercising individuals through a three-dimensional structure and entering the literature as a context-sensitive measurement tool for sport sciences, health sciences, and sleep research [1,2].

## 5. Conclusions

The EFA and CFA results support the structural validity of the scale; Cronbach’s alpha, McDonald’s omega, and CR values support internal consistency; however, AVE values below 0.50 indicate that convergent validity is only partially supported; and the Fornell–Larcker criterion provides evidence of discriminant validity. High effect sizes related to item discrimination show that the scale reliably distinguishes between individuals with low and high levels of exercise-context sleep quality. These findings indicate that ECSQS can be used in both research and applied settings and that it provides a context-sensitive assessment tool for studies on the exercise–sleep relationship. This study tested the ECSQS on a convenience sample of adults who exercise regularly in Osmaniye; while this provides valuable initial evidence, the findings may be limited in their generalizability to other Turkish communities or clinical groups. Also, test–retest reliability was not examined and it was not compared with commonly used sleep quality tools. These points do not represent shortcomings of the current study, but rather areas where further research is needed. Future studies are encouraged to use longitudinal designs to strengthen the evidence base for the ECSQS, to evaluate test–retest reliability, and to explore cross-cultural and criterion validity.

## 6. Recommendations

The scale should be used together with exercise type (aerobic, strength, combined), frequency, intensity, and duration, and the relationships between ECSQS scores and these parameters should be examined using longitudinal and experimental designs. In this way, the causal perception of exercise-related sleep and possible dose–response relationships can be clarified more clearly. ECSQS can be used as both a primary and secondary outcome measure in intervention studies evaluating the effects of exercise programs (e.g., 8–12-week aerobic or resistance exercise protocols) on sleep quality. Thus, the effects of such programs on sleep quality can be assessed in a context-specific manner. Adapting ECSQS into English and other languages will allow comparative investigation of sleep quality among exercising individuals in different countries. In this process, forward–backward translation, cultural adaptation, and measurement invariance analyses with multi-group CFA should be conducted [24]. Scale scores can be evaluated together with training load, recovery processes, and performance variables, providing an opportunity for early intervention in sleep disturbances. ECSQS items can be integrated into mobile health (mHealth) and exercise-tracking applications so that users receive regular feedback on the exercise–sleep relationship. This integration may be beneficial for both self-monitoring and behavior-change programs.

## Figures and Tables

**Table 1:** Distribution of Participants’ Demographic and Exercise-Related Characteristics According to EFA and CFA Samples.

	EFA (*n* = 310) Frequency (*n*)/%	CFA (*n* = 310) Frequency (n)/%
Gender		
Female	138/44.5	156/50.3
Male	172/55.5	154/49.7
Marital Status		
Single	251/81.0	250/80.6
Married	59/19.0	60/19.4
Education Level		
High school	33/10.6	45/14.5
Undergraduate	250/80.06	34/11.0
Postgraduate	27/8.7	231/74.5
What is the type of exercise you do most frequently?		
Aerobic	94/30.3	78/25.2
Combined	115/37.1	109/35.2
Strength	101/32.6	123/39.7

**Table 2:** Exploratory Factor Analysis Results for Exercise-Context Sleep Quality Scale (ECSQS) (*n* = 310).

Item	Factor 1	Factor 2	Factor 3	Common Variance (h^2^)
Factor 1.				
14—On the days I exercise, it’s easier for me to fall asleep.	0.70	–	–	0.52
15—On the days I exercise, I wake up feeling more refreshed.	0.75	–	–	0.59
16—Exercise generally has a positive effect on the quality of my sleep.	0.81	–	–	0.69
17—It takes me longer to fall asleep after exercising.	0.65	–	–	0.44
18—Aerobic exercise has a more positive effect on my sleep quality.	0.70	–	–	0.51
19—Strength training has a more positive effect on my sleep quality.	0.72	–	–	0.53
Eigenvalue	3.73			
Explained Variance (%)	31.07			
Factor 2.				
12—Noise negatively affects the quality of my sleep.	–	0.77	–	0.60
13—I can’t sleep without the light on.	–	0.75	–	0.53
8—Light negatively affects the quality of my sleep.	–	0.71	–	0.60
Eigenvalue		1.70		
Explained Variance (%)		14.23		
Factor 3.				
4—When I wake up in the morning, I feel well-rested.	–	–	0.80	0.67
1—My sleep quality is generally good.	–	–	0.80	0.65
6—My sleep schedule is regular.	–	–	0.65	0.47
Eigenvalue			1.38	
Explained Variance (%)			11.50	
Total Explained Variance (%)			56.8	

**Table 3:** Item Analysis and Internal Consistency Reliability.

Item	Corrected Item-Total Correlation	Alpha When Item is Deleted
F1		
ECSQS14	0.60	0.80
ECSQS15	0.63	0.80
ECSQS16	0.71	0.77
ECSQS17	0.52	0.82
ECSQS18	0.56	0.80
ECSQS19	0.58	0.80
F1—Cronbach Alpha = 0.83|McDonald Omega (ω) = 0.83|Number of items = 6		
F2		
ECSQS12	0.52	0.45
ECSQS13	0.47	0.51
ECSQS8	0.37	0.64
F2—Cronbach Alpha = 0.65|McDonald Omega (ω) = 0.65|Number of items = 3		
F3		
ECSQS4	0.48	0.51
ECSQS1	0.46	0.53
ECSQS6	0.42	0.60
F3—Cronbach Alpha = 0.64|McDonald Omega (ω) = 0.64|Number of items = 3		
Total Scale (12 items)—Cronbach Alpha = 0.77|McDonald Omega (ω) = 0.77		

**Table 4:** Goodness-of-Fit Indices.

Compatibility Index	Value	Limit Value	Source
χ^2^/df (CMIN/DF)	2.15	≤5.00	Kline [20]
RMR	0.05	0.05–0.08	Hooper et al. [24]
GFI	0.94	≥0.85/preferentially ≥0.90	Kline [20]
AGFI	0.91	≥0.80	Hooper et al. [24]
NFI	0.94	≥0.90	Hu & Bentler [24]
IFI	0.94	≥0.90	Hooper et al. [24]
TLI	0.92	≥0.90	Hooper et al. [24]
CFI	0.94	≥0.90	Hu & Bentler [25]
RMSEA	0.06	≤0.08	Hu & Bentler [25]

Note: The table only shows the fit indices found in the pasted AMOS output.

**Table 5:** Confirmatory Factor Analysis Fit Indices (ML Estimation, N = 310).

Model	χ^2^ (df)	CFI	TLI	RMSEA [90% CI]	SRMR
Default model	109.54 (51)	0.937	0.919	0.061 (0.045–0.077)	0.069
Independence model	1001.62 (66)	0.000	0.000	0.214 (0.203–0.226)	0.369
Saturated model	0.000 (0)	1.000	1.000	0.000	0.000

Note. Fit indices are based on maximum likelihood estimation. Bootstrap procedures with 200 resamples were applied to obtain robust standard errors and bias-corrected confidence intervals.

**Table 6:** Factor Loadings Findings.

Factor	Item	B	Standard Error	Critical Ratio	*p*	Standard Load
F1	ECSQS14	0.950	0.114	9.524	<0.001	0.681
F1	ECSQS15	1.029	0.124	10.188	<0.001	0.750
F1	ECSQS16	1.004	0.124	10.764	<0.001	0.827
F1	ECSQS17	0.961	0.129	8.098	<0.001	0.551
F1	ECSQS18	0.943	0.108	8.404	<0.001	0.577
F1	ECSQS19	1.000				0.616
F2	ECSQS 12	1.043	0.237	6.016	<0.001	0.772
F2	ECSQS 13	0.981	0.274	5.594	<0.001	0.613
F2	ECSQS 8	1.000				0.568
F3	ECSQS 4	0.978	0.154	6.427	<0.001	0.668
F3	ECSQS 1	1.132	0.163	6.463	<0.001	0.612
F3	ECSQS 6	1.000				0.566

**Table 7:** Differentiation of scale scores according to the upper and lower 27% groups.

Item	Item-Total Correlation	Bottom 27% Group (*n* = 84)	Top 27% Group (*n* = 84)	Factor	t	*p*	Cohen’s d	Effect Size
		Mean	Mean					
ECSQS14	0.60 **	1.91	3.52	1. Factor	−11.74	*p* < 0.001 *	−1.65	Very large
ECSQS15	0.63 **	1.75	3.48		−12.32	*p* < 0.001 *	−1.72	Very large
ECSQS16	0.66 **	1.68	3.49		−14.27	*p* < 0.001 *	−1.80	Very large
ECSQS17	0.54 **	1.98	3.69		−9.32	*p* < 0.001 *	−1.40	Very large
ECSQS18	0.56 **	1.60	2.94		−8.82	*p* < 0.001 *	−1.05	Big
ECSQS19	0.59 **	1.48	3.01		−10.40	*p* < 0.001 *	−1.28	Very large
ECSQS 4	0.40 **	2.00	3.31	2. Factor	−6.59	*p* < 0.001 *	−0.94	Big
ECSQS 1	0.50 **	1.84	3.38		−9.31	*p* < 0.001 *	−1.36	Very large
ECSQS 6	0.40 **	2.38	3.48		−5.87	*p* < 0.001 *	−0.87	Big
ECSQS12	0.40 **	2.45	3.55	3. Factor	−7.06	*p* < 0.001 *	−1.04	Big
ECSQS8	0.49 **	2.30	3.54		−8.60	*p* < 0.001 *	−1.19	Big
ECSQS13	0.48 **	3.31	4.71		−8.98	*p* < 0.001 *	−1.25	Very large

* moderate significance, ** highly significant.

**Table 8:** Descriptive Statistics for the Factors and Total Scale.

	N	Mean	Standard Deviation	Minimum	Maximum	Scale Min., Max.
FACTOR 1: Perception of Exercise-Related Sleep	310	14.48	5.29	6.00	30.00	12–60
FACTOR 2: Sleep Hygiene and Environmental Factors	310	7.86	3.05	3.00	15.00	
FACTOR 3: General Sleep Adequacy	310	9.73	2.67	3.00	15.00	
ECSQS Total	310	32.07	7.89	12.00	60.00	

**Table 9:** Item Reduction Process.

Item	Stage	Factor Loading	Cross-Loading	Communality	Corrected Item–Total Correlation	Decision	Reason for Deletion
ESCQS2	Stage 1	–	–	–	<0.30	Deleted	Low item–total correlation
ESCQS3	Stage 1	–	–	–	<0.30	Deleted	Low item–total correlation
ESCQS5	Stage 1	–	–	–	–	Deleted	Content overlap
ESCQS7	Stage 1	–	–	–	–	Deleted	Content overlap
ESCQS9	Stage 2	<0.40	–	–	–	Deleted	Factor loading below 0.40
ESCQS10	Stage 2	<0.40	–	–	–	Deleted	Factor loading below 0.40
ESCQS11	Stage 2	–	Cross-loading	–	–	Deleted	Cross-loading on multiple factors
ESCQS20	Stage 2	–	Cross-loading	–	–	Deleted	Cross-loading on multiple factors
ESCQS21	Stage 2	–	Cross-loading	–	–	Deleted	Cross-loading on multiple factors

## Data Availability

The study data can be obtained by contacting the author of correspondence, Mustafa Can Koc, at cankoc_01@hotmail.com.
